# Supplementary material for: Plasmodium vivax genomic surveillance in the Peruvian Amazon with Pv AmpliSeq assay
Source: PLoS Negl Trop Dis. 2024 Jul 11;18(7):e0011879. doi: 10.1371/journal.pntd.0011879 (PMC11265702; doi:10.1371/journal.pntd.0011879)
Supplement: S1 Text — Fig A. Flowchart describing number and type of samples and control attempted with the Pv AmpliSeq assay in this study. Fig B. Global Plasmodium vivax population structure. First two principal components of PCA analysis of 1474 high-quality P. vivax genomes using LD-pruned SNPs across the core genome as previously described in Kattenberg et al 2024 (https://doi.org/10.1002/ece3.11103). The samples (dots) are colored according to the originating population (here region), following classifications from Adam et al., 2022 (https://doi.org/10.12688/WELLCOMEOPENRES.17795.1). Fig C. Distribution of depth of coverage for each amplicon in the Pv AmpliSeq v2 Peru assay. Fig D. Proportion of polyclonal infections detected in each district. Within-host infection complexity was used as a measure of complexity of infections, using within-sample F-statistic (Fws) ≥ 0.95 as proxy for a monoclonal infection. Fig E. Scatter plot of principal components 1 & 2 (A) and 3 & 4 (B) using all variants detected by the Pv AmpliSeq v2 Peru assay of Peru samples (n = 230) grouped by district. Fig F. Parasite relatedness. Network of individual relatedness at (A) intermediate levels of relatedness (50% IBD threshold) and (B) Very high levels of relatedness, indicating clonal infections (95% IBD threshold) colored by years. Fig G. Parasite relatedness and haplotypes of variants of interest. Network of individual relatedness at intermediate levels of relatedness (50% IBD threshold) colored by haplotypes for the different genes. Fig H. Distribution of pvama1 haplotypes by year and district. (DOCX) [file pntd.0011879.s007.docx]

# **S1 Supplementary figures**


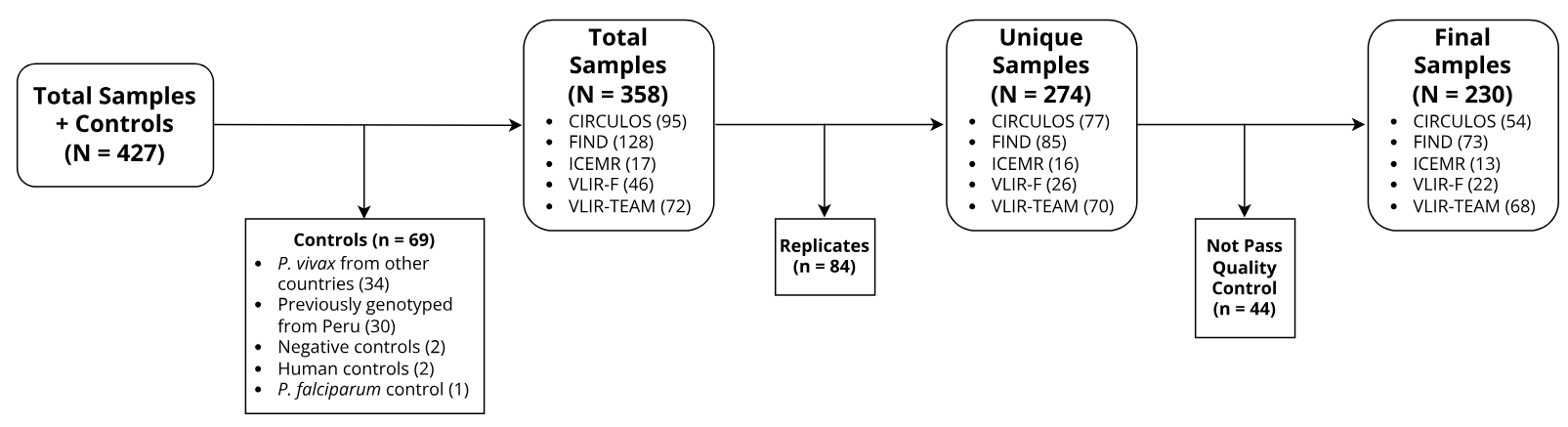


**Fig. A. Flowchart describing number and type of samples and control attempted with the Pv AmpliSeq assay in this study.**


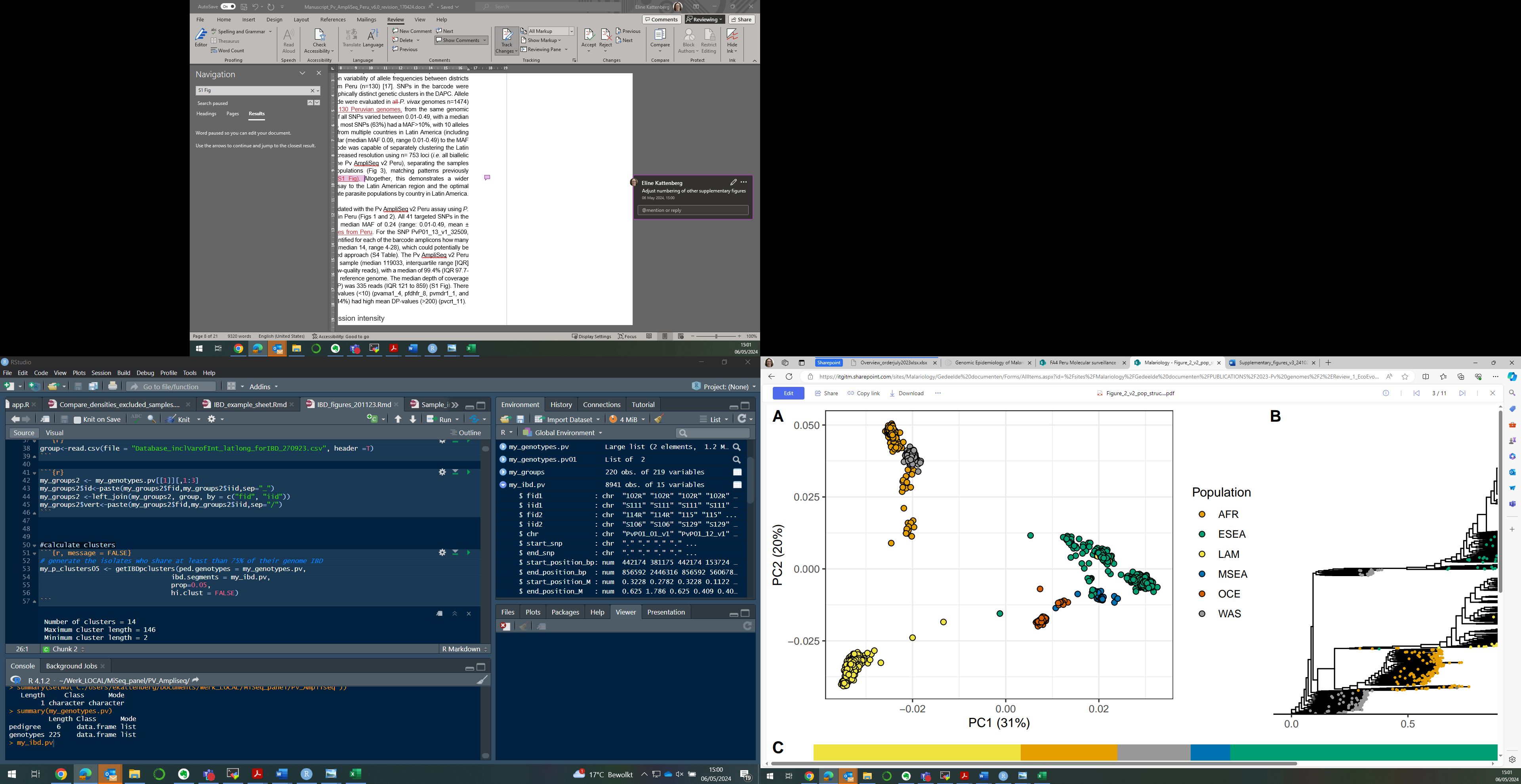


**Fig. B. Global *Plasmodium vivax* population structure.** First two principal components of PCA analysis of 1474 high-quality *P. vivax* genomes using LD-pruned SNPs across the core genome as previously described in Kattenberg et al 2024 (<https://doi.org/10.1002/ece3.11103>). The samples (dots) are colored according to the originating population (here region), following classifications from Adam et al., 2022 (<https://doi.org/10.12688/WELLCOMEOPENRES.17795.1>).

**
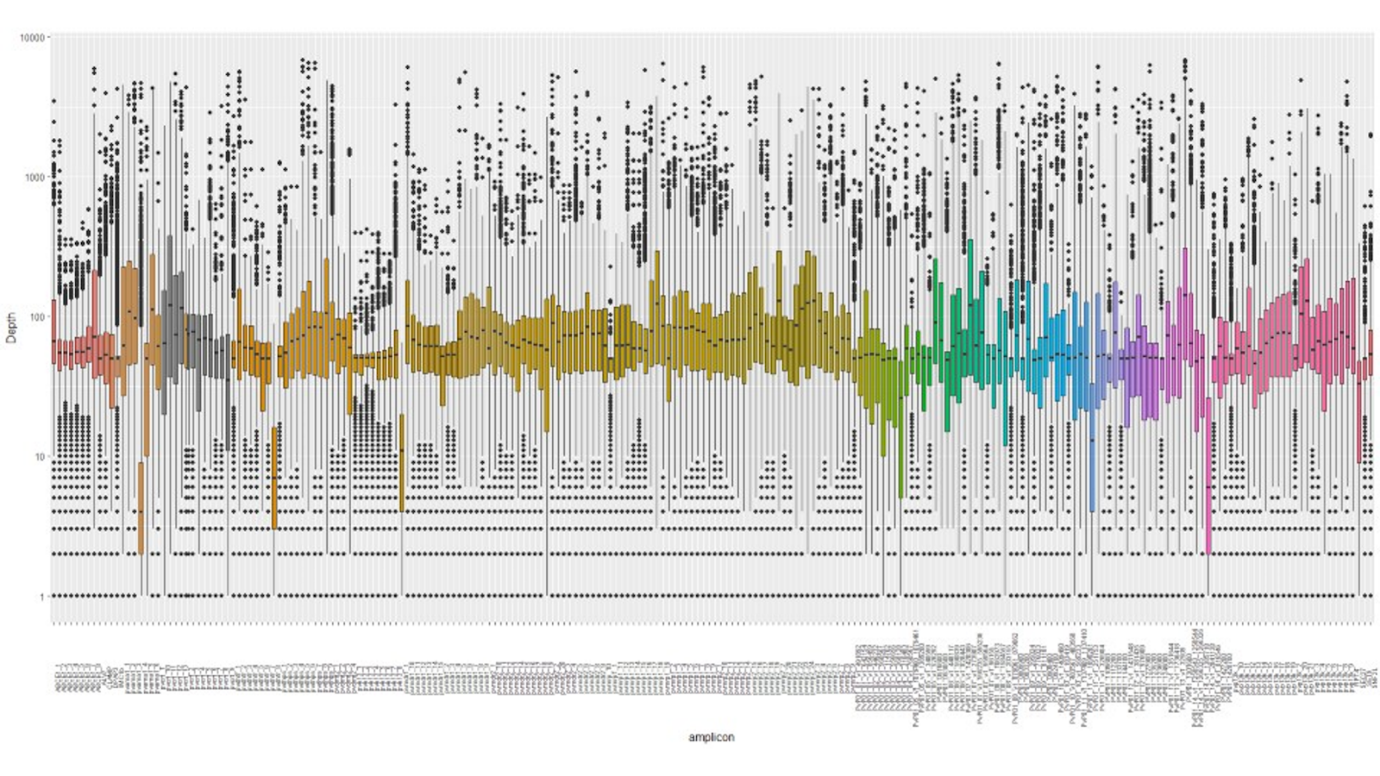
**

**Fig. C. Distribution of depth of coverage for each amplicon in the Pv AmpliSeq v2 Peru assay.**


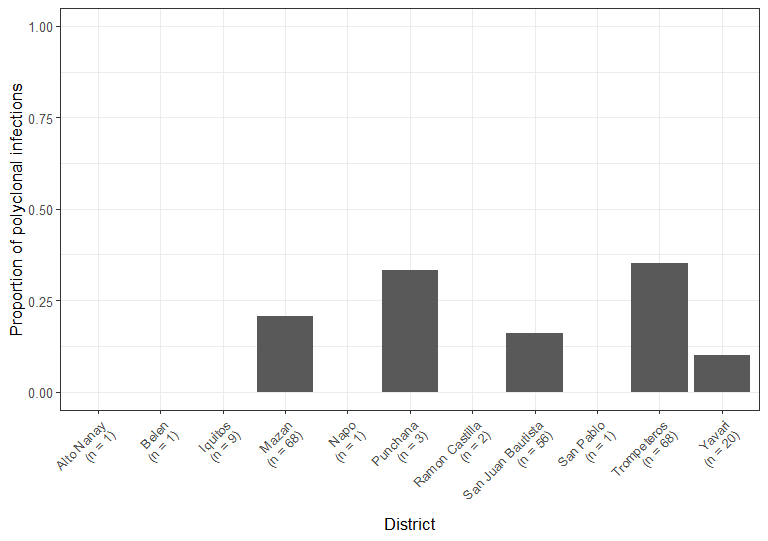


**Fig. D. Proportion of polyclonal infections detected in each district.** Within-host infection complexity was used as a measure of complexity of infections, using within-sample F-statistic (Fws) ≥ 0.95 as proxy for a monoclonal infection.


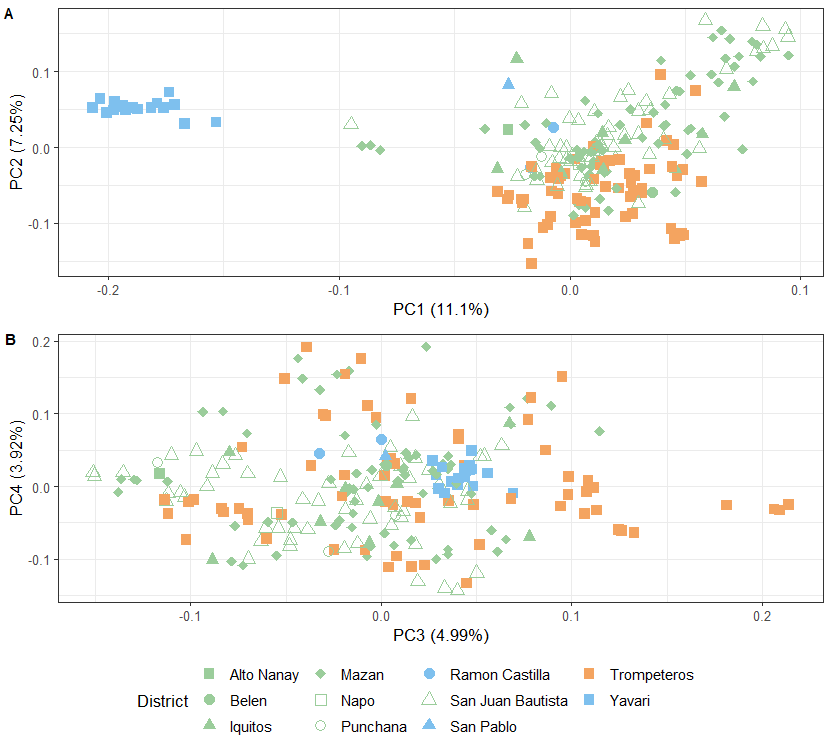
 **Fig. E. PCA - Scatter plot of principal components 1 & 2 (A) and 3 & 4 (B) using all variants detected by the Pv AmpliSeq v2 Peru assay of Peru samples (n = 230) grouped by district.**

**
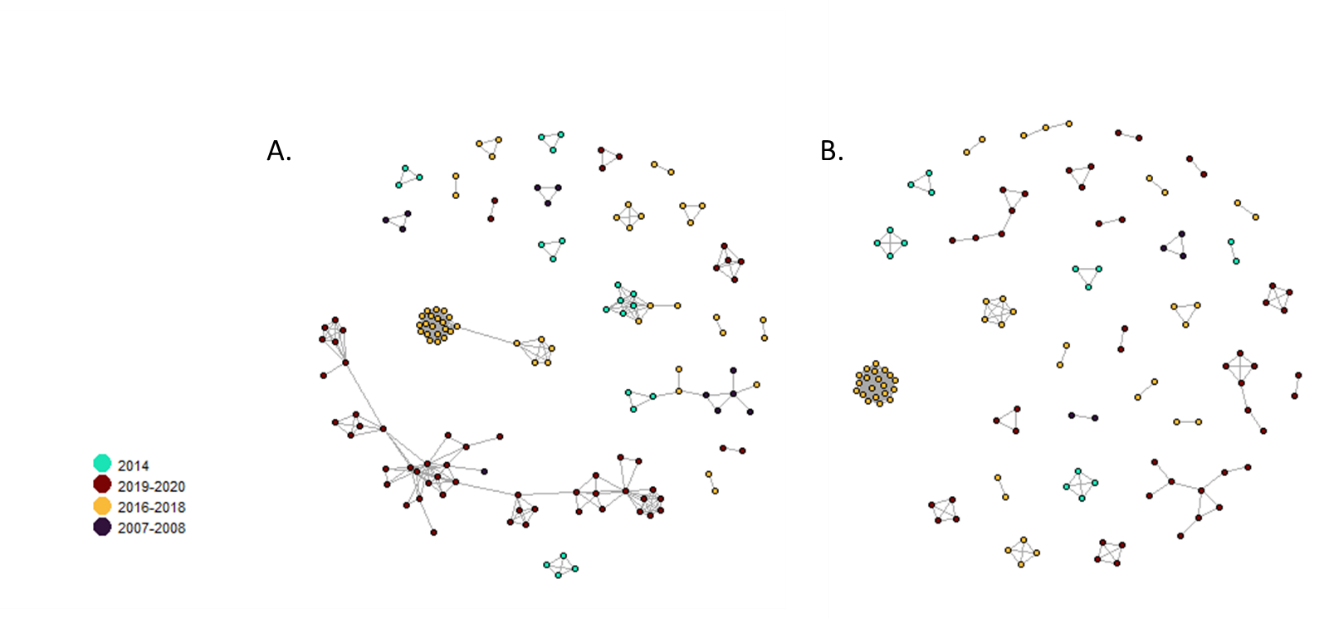
**

**Fig. F. Parasite relatedness.** Network of individual relatedness at (A) intermediate levels of relatedness (50% IBD threshold) and (B) Very high levels of relatedness, indicating clonal infections (95% IBD threshold) colored by years.


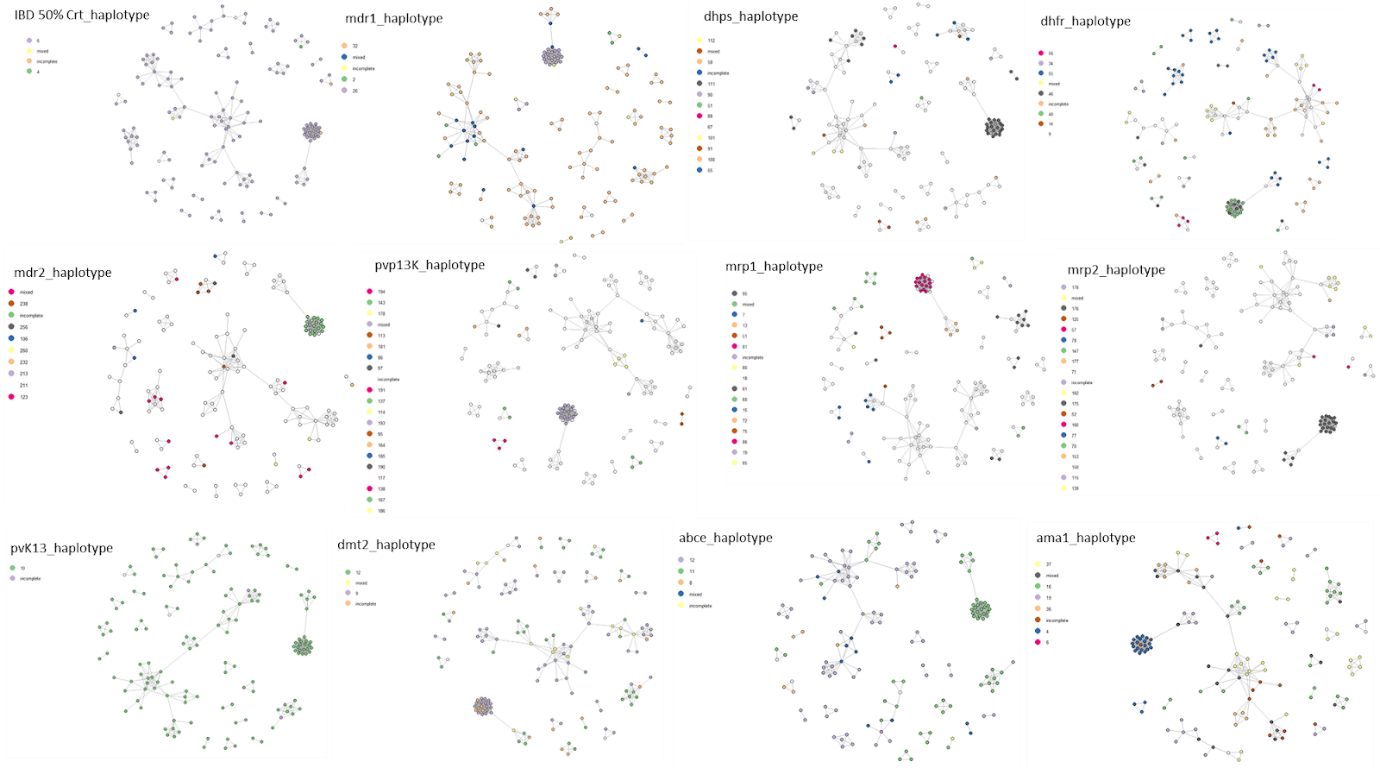
 **Fig. G. Parasite relatedness and haplotypes of variants of interest.** Network of individual relatedness at intermediate levels of relatedness (50% IBD threshold) colored by haplotypes for the different genes.


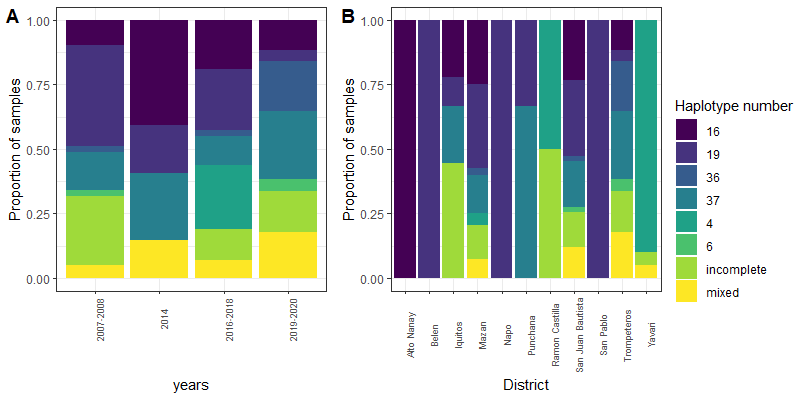
 **Fig. H. Distribution of *pvama1* haplotypes by year and district.**
