# Supplementary material for: Plasmodium vivax genomic surveillance in the Peruvian Amazon with Pv AmpliSeq assay
Source: PLoS Negl Trop Dis. 2024 Jul 11;18(7):e0011879. doi: 10.1371/journal.pntd.0011879 (PMC11265702; doi:10.1371/journal.pntd.0011879)
Supplement: Attachment — Submitted filename: Reviewer_comments3.doc [file pntd.0011879.s009.doc]

Reviewer 1 report

**Plasmodium vivax genomic surveillance in the Peruvian Amazon with Pv AmpliSeq**

**Assay**

This manuscript by Johanna Helena Kattenberg and colleagues examined Plasmodium vivax genomic surveillance in the Peruvian Amazon using multiplex and robust Pv AmpliSeq panel. The study is timely study as Pv highly prevalent in hard to reach community of Peru and it is a major impediment to achieve malaria elimination goals. Overall, the manuscript is well written however it lacks novelty and key gaps to be addressed by the study. Detecting previously reported population differentiation is not good enough.

**Methods**

Number of samples you attend vs successfully genotyped not mentioned. This is very important to see your new Pv AmpliSeq panel robustness. Moreover, the following statement “For the analysis of genomic surveillance use cases in Peru, we included 230 samples with good quality data (<25% missing genotype calls for all variants, mean coverage >15), and retained only one library of replicates (with lowest missingness)” not clear. What is minimum sample and SNPs missingness rate in your data set. How many of samples and SNPs/loci/genotypes removed from total based on your filtering criteria. The association between sequence coverage and parasite density is critical but not mentioned!

**Peru SNP selection criteria**

Any specific SNPs contributing for the observed parasite population clustering by DAPC? I would expect loci under selection or nearby drug resistance might contributed more for observed pattern of parasite clustering. So, should how neutral or relatively neutral selected SNPs? Otherwise the SNPs and analysis violates basic population genetic principles? Markers should be neutral or not under selection.

**DAPC**

The cluster method specially DAPC over estimates clustering as it maximum difference between population and minimize difference with populations. So, I urge the authors redo cluster analysis using classical PCA or/AND t-SNES and compare it with DAPC output.

**IBD**

The author used isoRelate package in R which was designed for WGS data and more accurate when you have genome-wide loci (1000s plus loci). Therefore, it is not appropriate to use isoRelate default setting this data set (very few loci across genome). It is critical to mention what modification made not to report false IBD. I also request to use other available packages compare the IBD finds from isoRelate software.

**Statistics**

For this “Moderate levels of *He* were found in all districts, except in Yavari, which

had a significantly lower diversity (mean *He* = 0.0121; p < 0.0001) (Fig 4A)” and other statistics it is worthy do sample size adjustment and weighing? The sample size per year or site variation is huge and some sites have very few samples (n=2). Also Prediction of origin of *P.vivax not cleared stated in statistic and method part and I am not sure how authors draw figure 7. So it is worth to explicitly mention how* Prediction of origin of *P.vivax made.*

**Minor comments**

1. MAF calculation vs sample size per population? Did you adjust sample size variations as MAF significantly affected by sample size per population?

2. Comparing your 41 targeted SNPs vs global Pv barcode, microsatellites or WGS to see how your newly developed barcode is robust enough to differentiate local parasite from global

3. FST vs IBD sharing and HE VS FWS. Worthy to mention which metrics are robust for Pv genomic surveillance and more correlated with transmission intensity.

4. All figure resolution is not good as it is. Please consider submitting high resolution figs for final submission.

5. What is limitation of your study? Sample size?
