# Supplementary material for: Plasmodium vivax genomic surveillance in the Peruvian Amazon with Pv AmpliSeq assay
Source: PLoS Negl Trop Dis. 2024 Jul 11;18(7):e0011879. doi: 10.1371/journal.pntd.0011879 (PMC11265702; doi:10.1371/journal.pntd.0011879)
Supplement: Attachment — Submitted filename: Reviewer_comments_and_responses_V2.docx [file pntd.0011879.s010.docx]

Dear dr. Andrés Aranda-Diaz, dr. Susan Madison-Antenucci and reviewers,

We hope this letter finds you well. We are writing to submit a revised version of our manuscript titled "Plasmodium vivax genomic surveillance in the Peruvian Amazon with Pv AmpliSeq assay". We sincerely appreciate the thoughtful feedback provided by the reviewers and the editorial team during the initial review process.

In response to the reviewers' comments, we have carefully revised the manuscript to address their concerns and improve the clarity of our work. Below, we summarize the major revisions made in response (black text) to each reviewer's feedback (in blue text). Line numbers refer to the document version that has the tracked changes.

**Reviewer 1**

1. This manuscript by Johanna Helena Kattenberg and colleagues examined Plasmodium vivax genomic surveillance in the Peruvian Amazon using multiplex and robust Pv AmpliSeq panel. The study is timely study as Pv highly prevalent in hard to reach community of Peru and it is a major impediment to achieve malaria elimination goals. Overall, the manuscript is well written however it lacks novelty and key gaps to be addressed by the study. Detecting previously reported population differentiation is not good enough.

We thank the reviewer for the comments on the manuscript. We have made modifications to the manuscript to emphasize the novelty and make it more clear (specifically in lines 122-140, and other clarifications throughout the manuscript).

Most importantly, we have designed a novel SNP barcode specifically for MMS in Peru. This is a new barcode, for which new primers were designed in order to incorporate it into an NGS assay (i.e. modification of a AmpliSeq assay previously reported). We have applied the modified assay, with the new barcode, to samples from Peru collected between 2007-2020. The analysis presented with these samples in this manuscript is new, and previous studies including these samples did not report population genetic analysis nor drug resistance surveillance data. In addition, we analyze for the first time, isolates from remote communities (e.g. Yavari and Trompeteros) that have previously not been studied in Peru, which uncovered new insights on the population structure of *P. vivax* in these communities.

Only in the first analysis presented in the results (including Fig. 3) in lines 292-312, we make a comparison of the population structure and differentiation of the barcode SNPs vs a larger set of biallelic SNPs in previously reported samples and data. The aim of this section is to validate the new barcode (named Pv Peru-barcode) *in silico* in previously analyzed samples and therefore we demonstrate that it can “detect previously reported population differentiation”. Besides this *in silico* analysis, in the rest of the manuscript we present novel data by applying the Pv AmpliSeq v2 assay on new samples. .

2. Number of samples you attend vs successfully genotyped not mentioned. This is very important to see your new Pv AmpliSeq panel robustness. Moreover, the following statement “For the analysis of genomic surveillance use cases in Peru, we included 230 samples with good quality data (<25% missing genotype calls for all variants, mean coverage >15), and retained only one library of replicates (with lowest missingness)” not clear. What is minimum sample and SNPs missingness rate in your data set. How many of samples and SNPs/loci/genotypes removed from total based on your filtering criteria.

We agree with the reviewers that this information is important and have added more details in lines 229-233 and added a flowchart of sample exclusion steps as S1 Fig.

We have filtered variants (SNPs and indels) on the criteria reported in lines 223-224: “Variants were hard filtered (QUAL>30, overall DP>100, MQ>50, QD>1.0, SOR<4, GT depth >5) and annotated with SnpEff (v4.3T) [38], resulting in 3862 high quality genotypes (incl. all variant types, *e.g.* SNPs and indels).” We did no further filter on SNP missingness rate for the SNPs in general. Only for the IBD analysis did we apply additional filtering for the variants, which was added in lines 275-283.

We did apply a missingness filter for the samples, by removing samples from the analysis, which were missing more than 25% genotype calls (SNPs and indels). In addition, we removed samples that had a mean depth of coverage lower than 15 reads. Some samples were run in duplicate or triplicate, in which case, we retained only one replicate for the final genetic analyses. This information has now been added in a flow chart as supplementary figure S1 Fig.

3. The association between sequence coverage and parasite density is critical but not mentioned!

While indeed the parasite density has an impact on the sequence coverage, this predominantly occurs at very low parasite densities. The relationship parasite density vs sequencing coverage is not linear, as we dilute and pool libraries at the same concentration (2nM each) prior to sequencing (line 213). In other words, at high enough parasite densities, we get similar depth of coverage (as can be seen in Fig S2), because the libraries are equilibrated. We apply a CT value/density cutoff for sample selection in order to include samples that will lead to good library quantity and thus depth of coverage. Samples below the cutoff are excluded. Definition of cutoffs by parasite densities was described in detail in our previous manuscript (Kattenberg et al. 2023, <https://doi.org/10.3389/fcimb.2022.953187>). We clarified this in the methods section in lines 172-174.

4. Peru SNP selection criteria –
   a. Any specific SNPs contributing for the observed parasite population clustering by DAPC?

Yes, this is part of how we select the SNPs as described in lines 156-162.

We also observe that SNPs in the barcode contribute to the observed population clustering in the Peruvian samples, as explained in lines 385-390 and listed in S5 Table.

b. I would expect loci under selection or nearby drug resistance might contributed more for observed pattern of parasite clustering. So, should how neutral or relatively neutral selected SNPs? Otherwise the SNPs and analysis violates basic population genetic principles? Markers should be neutral or not under selection.

Before performing the DAPC, we selected SNPs on all chromosomes and did not allow any SNPs closer than 500 bp to drug resistant amplicons. 500 bp is the average distance of linkage disequilibrium decay in *Plasmodium*, therefore we do not expect these loci to be under selection. In addition, there is no evidence of strong selection on drug resistance genes in *P. vivax* populations in general. The SNP barcode amplicons are spread across all chromosomes, at large distance from each other, and therefore expected to be unlinked. Besides this, with the advancement of NGS approaches and feasibility of screening large amount of markers, there is a growing awareness that adaptive markers can be of added value to molecular ecology and population genetic studies (see <https://www.ncbi.nlm.nih.gov/pmc/articles/PMC3131602/> for example).

5. DAPC - The cluster method specially DAPC over estimates clustering as it maximum difference between population and minimize difference with populations. So, I urge the authors redo cluster analysis using classical PCA or/AND t-SNES and compare it with DAPC output.

As we are interested in the differentiation between populations (confirmed in a first step in the Fst analysis), and the variants contributing to the differentiation patterns, we believe the DAPC approach is appropriate. We use a cross-validated DAPC approach to avoid overfitting the data (as recommended in https://doi.org/10.1186/1471-2156-11-94). DAPC is commonly applied in malaria molecular studies, including our own ( see for example: <https://www.nature.com/articles/s41467-023-39417-1>, <https://doi.org/10.3389/fepid.2023.1227071>, <https://www.nature.com/articles/s41598-022-21028-3>, <https://doi.org/10.1186%2Fs13073-018-0563-0> )

PCA was performed in initial data browsing and analysis, but not previously included in the manuscript. We have added it now as supplementary figure S5, and it shows similar clustering patterns as observed with DAPC, with the Yavari population differentiation responsible for the highest amount of variation. The DAPC is better at separating Trompeteros from Maynas populations, and the contributing variants to this differentiation can be investigated with the allele loadings in the DAPC, therefore we prefer to keep this analysis.

However, we would like to note that PCA also has its drawbacks as described here: Elhaik, E. Principal Component Analyses (PCA)-based findings in population genetic studies are highly biased and must be reevaluated. Sci Rep 12, 14683 (2022). <https://doi.org/10.1038/s41598-022-14395-4>. But likely, both tSNE and DAPC are sensitive to similar artifacts.

6. IBD - The author used isoRelate package in R which was designed for WGS data and more accurate when you have genome-wide loci (1000s plus loci). Therefore, it is not appropriate to use isoRelate default setting this data set (very few loci across genome). It is critical to mention what modification made not to report false IBD. I also request to use other available packages compare the IBD finds from isoRelate software.

We used the isoRelate package for IBD analysis using 1219 SNPs, and not just the SNP barcode loci; this was clarified in lines 275-285. Taylor et al reported that 200 biallelic markers are sufficient for IBD analysis (<https://doi.org/10.1534/genetics.119.302120>), as pointed out by reviewer nr 3. We added a description of the reasons for the different subsets of variants used in lines 235-249 for clarification.

We believe the approach used with the nr of SNPs and IsoRelate package is appropriate for the purpose of investigating connectivity between districts and clustering patterns in and between these districts.

We did not use the default settings, but adjusted settings as described in Kattenberg JH, Nguyen HV, Nguyen HL, Sauve E, Nguyen NTH, Chopo-Pizarro A, et al. Novel highly-multiplexed AmpliSeq targeted assay for Plasmodium vivax genetic surveillance use cases at multiple geographical scales. Front Cell Infect Microbiol. 2022;12:953187. Epub 2022/08/30. doi: 10.3389/fcimb.2022.953187. To clarify this we have added the details of the used settings to the methods in lines 277-283.

7. Statistics - For this “Moderate levels of He were found in all districts, except in Yavari, which

had a significantly lower diversity (mean He = 0.0121; p < 0.0001) (Fig 4A)” and other statistics it is worthy do sample size adjustment and weighing? The sample size per year or site variation is huge and some sites have very few samples (n=2).

Statistical tests (Kruskal-Wallis and Pairwise Willcoxon with correction) were added to the manuscript in lines 254-259. To avoid issues due to low sample size, for the comparison by place, districts with n <10 were grouped as “Others” (n = 7). To compare by time, we grouped into 4 periods: 2007-08 (n=48), 2014 (n=35), 2016-18 (n=199) and 2019-20 (n=83).

8. Also Prediction of origin of *P. vivax* not cleared stated in statistic and method part and I am not sure how authors draw figure 7. So it is worth to explicitly mention how Prediction of origin of P.vivax made.

We used a published likelihood model for the prediction of origin using the 33-SNP vivaxGEN-geo barcode and accompanying tool, as explained in lines 287-289: “A likelihood-based classifier was used to predict the origin of P. vivax isolates using the 33-SNP vivaxGEN-geo barcode, using the vivaxgen geo framework (https://geo.vivaxgen.org/) and reference dataset [44]”. We adjusted the caption of figure 7 to clarify the model that was used and what frequency is plotted in the figure in lines 981-988 to help with the interpretation of the figure.

9. Minor comments

1. MAF calculation vs sample size per population? Did you adjust sample size variations as MAF significantly affected by sample size per population?

MAF of the SNPs was not reported per population within Peru. This was clarified in line 317.

2. Comparing your 41 targeted SNPs vs global Pv barcode, microsatellites or WGS to see how your newly developed barcode is robust enough to differentiate local parasite from global

The designed 41 SNP barcode is compared to a larger set of SNPs (from the WGS data) in the PCA in figure 3. The PCA with all SNPs detected (n=1,477,945 SNPs) in the WGS has a higher resolution than the SNP barcode as can be expected from the higher number of SNPs, but shows similar clustering patterns at the global level (see supplementary Fig S2 with the PCA of all WGS SNPs that was added). Comparison of the barcode to MS data was not part of this study, but is being done in an additional study and will be included in a separate publication.

Besides, we use the previously published 33-SNP Vivaxgengeo barcode to do the prediction of origin at a global level. The prediction might be improved by including the 41-SNPs to the likelihood model, which does not perform that well in this region (reported in Kattenberg et al 2023). However, evaluating and adjusting the likelihood model was not the objective of the current study.

3. FST vs IBD sharing and HE VS FWS. Worthy to mention which metrics are robust for Pv genomic surveillance and more correlated with transmission intensity.

We agree with the reviewer that these are interesting correlations to investigate and not well established for *P. vivax* populations (in Peru and in general), as is also discussed here <https://doi.org/10.1179/2047773215y.0000000012> for example. However, we do not feel that we can make appropriate conclusions on this matter based on the data presented in this manuscript.

4. All figure resolution is not good as it is. Please consider submitting high resolution figs for final submission.

Figure resolution and format are submitted as per the journal requirements.

5. What is limitation of your study? Sample size?

Limitations are discussed in the discussion lines 562-603. In summary this is indeed the limited number of study sites, therefore we are not able to give a full representation of the *P. vivax* population in all endemic districts in Peru, which would be a task for routine surveillance. Another limitation is the lack of validated markers of resistance to the drugs used to treat *P. vivax.*

**Reviewer #2: Methods comments:**

10. What was the success rate? You mention 230 samples, but how many did you attempt to use initially?

We agree with the reviewers that this information is important and have added this in lines 229-233 and S1 Fig. Our success rate was 84% (230/274).

11. I think you could add a few more words on the in-house variant calling pipeline. Is this a (targeted) mapping + GATK haplotype calling procedure as commonly applied to WGS reads, or are additional amplicon denoising strategies applied?

We used a procedure similar to WGS, and not additional amplicon denoising programs, additional details have been added to lines 219-220. All scripts can be found in the github link (<https://github.com/Ekattenberg/Plasmodium-AmpliSeq-Pipeline>) together with a description with the different steps in the pipeline.

12. Whats the target size range (excl adapters) and how many samples do you sequence in single 600-cycle run?

We have added the target sizes in the Table S2 and the size range of the targets in line 208. We usually multiplex 144 libraries in one run, this information was added to line 214.

13. Why do you select Fws ≥ 0.95 as a proxy for a monoclonal infection?

This is a commonly used threshold for monoclonal infections, as described in Auburn et al. 2012 <https://doi.org/10.1371/journal.pone.0032891>. We have now added the reference to this paper to make this clear in line 267.

14. Would it be useful to show sampling month in Fig 2 (e.g., as pie slices) and in S1 Table, since suggesting outbreak dynamics in some areas of text?

We have added the sampling by month in S1 Table.

15. Results comments:
    a. Fig. 1A: Could be expanded with larger labels and replace 1B,C,D - which currently provide little extra info.

Since text talks of peri-urban Pv connectivity (as opposed to simply spatial proximity-based connectivity) it would be useful to see some representation of urban extent (around Iquitos etc) in the map. Currently also the grey lines are not clearly explained. Rivers etc? Also, it could be useful to use some of the same province-level coloring in components of Figs 5-6? (since it seems most relatedness patterns related to within versus between province membership).

Fig. 1A has been updated following the suggestions. We added labels for the districts in Loreto and Ramon Castilla provinces. As districts in Maynas provinces are too close to each other to be added, we kept the map for this province as panel 1B. Gray lines show provinces and district borders in panels 1A and 1B, respectively.

b. Fig. 3: I suggest to label the genome/assay regions underlying each PCA directly in the subplots.

The labels of the SNP subset used in the subplots was added to figure 3.

c. Figs 5A-B: Currently the node colors are not visible enough. Could make them bigger and better associate coloring with geography, e.g., add a small map and/or associate coloring with main map in Fig. 1.

Colors of the nodes in Figure 5 was changed, now matching the colors of the districts in figure 1.

d. How are p-values calculated for differences in Pi for Iquitos compared to its surrounding districts and for 2007-2008 vs. later years?

Statistical tests (Kruskal-Wallis and Pairwise Willcoxon with correction) were added to the manuscript in lines 254-259.

e. Also please label Fig. 6A-B thresholds directly in the plot.

IBD thresholds were added to figure 6

f. Please clarify meaning of left vs. right in Fig. 6C. Thickness of curves on the left indicates how many samples from the left-indicated district show IBD > 0.10 IBD to any sample in the district indicated at right? Etc.

Clarification was added to the figure legend (see lines 976-978). The figures show pairwise IBD (sample x on the right vs sample y on the left), but summed by district.

16. Conclusions/Discussion comments:

I think a few sentences putting results in the context of transmission dynamics observed in other South American Pv regions would be useful. For example, the study suggests relatively clear regional clustering within Peru, signals of Brazilian diversity and outbreaking in Mariscal Ramon Castilla, and temporal turnover in Maynas. Do other regions show similar degrees of spatiotemporal population differentiation? If yes/no, how might environmental configuration and human activity patterns play a role?

We thank the reviewer for this suggestion. We have added a paragraph about transmission dynamics in several Latin American countries (Brazil, Colombia, Panamá and Venezuela) in lines 519-542.

**Reviewer #3:**

The article describes some great, new, country-specific (Peru) amplicon-based sequencing tools for P. vivax that have the potential to support control and elimination efforts in Peru. The methods, the data showcasing the utilities of the tools and the conclusions are mostly robust. I recommend a few revisions that may help to improve the clarity of various approaches. One of the major confusions is regarding whether one can/should use the 41 biallelic SNPs and when one needs to use the full panel of variants called. The abstract only refers to 41 biallelic barcode SNPs, but many analyses use a larger panel of markers. More clarification on what one can expect from the tools (41 or hundreds of SNPs?) would be helpful for the reader to better understand what they might expect to achieve in their own studies if they use these tools.

We thank the reviewer for the comments on the manuscript. We understand the confusion with the different subsets of SNPs from the AMpliSeq data and have tried to clarify this throughout the manuscript. In addition, we have specifically added our reflection on which SNP subset to use when in the methods in lines 235-249.

In summary, while the Pv Peru-Barcode is appropriate to be used for population genetic analyses, higher geographical resolution with the AmpliSeq assay data can be obtained by using all biallelic variants or SNPs in the vcf (n_loci_ = 3862, including n_SNP_ = 2909). Using all biallelic variants in the assay also allows the use of models such as identity-by-descent, which require a higher resolution/higher information content than the 41 SNPs. We use the biallelic SNPs as we previously saw these variants had a lowest error rate compared to indels.

Minor revisions

1. The introduction has an entire paragraph describing the challenge of asymptomatic vivax in Peru (p3), which gives the implication that the tools can help to address this. However, the Discussion section doesn't revisit how the study tools address this particular challenge. Some comments on this would be helpful.

We have added some sentences on the inclusion of asymptomatic infections in the Pv AmpliSeq assay, how important it is to consider these infections in molecular surveillance in Latin America lines 605-615.

2. The Introduction also alludes to the asymptomatic infections as a P. vivax-specific challenge - is that strictly true?

We agree that asymptomatic infections can be a challenge in itself, irrespective of the species. However, in the context of Peru and the Amazon region, asymptomatic infections are a common problem associated with *P. vivax* infections, as discussed for example in https://doi.org/10.1016%2Fj.lana.2021.100169, and less so with *P. falciparum*. In the introduction we also specifically mention that this is a challenge for “P. vivax infections in the region” (lines 80-81).

3. On p4, AmpliSeq tools are described as having previously identified temporal challenges before and after NMCP intervention (along with other utilities) - are the appropriate references for this statement 22 and 23?

The references were added to this statement in lines 119-121.

4. On p5, it would be helpful to better understand why the authors aimed for 49 SNPs? Is this purely because of the DAPC results or did they have a prior maximum number based on multiplex requirements with the other SNPs? Did they consider IBD requirements as per the specifications of Taylor and colleagues for 200 biallelic SNPs (https://pubmed.ncbi.nlm.nih.gov/31209105/)? Some further details would be helpful here.

We aimed for 3* 14 =42 SNPs (3 SNPs per chromosome), but we selected a few more for the design phase, knowing some could fail in this step. This was clarified in lines 160-161. For 8/49 we were unsuccessful to design primers that would work with the rest of the existing amplicons.

Being able to perform IBD analysis was not part of the consideration, as the barcode was designed before the IBD methods for plasmodia were published. However, in order to have sufficient number of biallelic SNPs for the IBD analysis, is why we include all biallelic SNPs, and not just the barcode SNPs detected in the samples. This has been clarified in lines 235-249 and 277-283.

5. On p6, the authors describe <25% missingness as the cut-off - across all variants if I recall correctly. What was the maximum observed missingness at the 41-SNP barcode in the 230 defined good-quality samples?

Indeed, samples with >25% missingness were excluded. Within the 230 good quality samples that were included in the analysis, 7 samples that missed all SNPs in the barcode. This was due to a human-error while performing the PCR, where we used the primers for the Pv AmpliSeq Vietnam assay ( https://doi.org/10.3389/fcimb.2022.953187) by error instead of the primers for the Peru-barcode described here. However, those samples were still included in the analysis because they had <25% missingness considering ALL variants in the AmpliSeq. These 7 samples contributed genetic data on the drug resistance markers analysis. The rest of the samples, 185 (80.4%) and 213 (92.6%) had <10% and <=25% missingness at the SNP barcode. This information was added in lines 319-325.

6. On p6, the authors refer to 230 high-quality samples - is this 100% (i.e. 230/230) of samples on which sequencing was attempted?

We agree with the reviewers that this information is important and have added this in lines 229-233 and S1 Fig. Our success rate was 84% (230/274).

7. On p7 the authors describe using Fws to characterize polyclonality - I believe on the 41-SNP barcode but they might clarify this. Given that the Fws was designed for genome-wide data, how well would this measure extrapolate to smaller barcodes?

The Fws analysis was performed on all biallelic SNPs (n=2909), this was clarified in line 266. We agree with the reviewer it would not be appropriate to use this approach with only the 41 SNPs.

To provide some context: We previously tested several methods to estimate MOI from barcodes and obtained highly different results depending on the method (see https://doi.org/10.1128/spectrum.00960-22 for example). Therefore, we conducted tests with various tools, including WGS and smaller datasets like the ~2000 SNPs detected in the ampliseq (unpublished data). From this analysis, we found that Fws provided robust estimates for complex infections, similar to MOI estimated by microsatellite data (unpublished data).

8. On p7, the authors refer to a genomic dataset comprising 1474 genomes - is this the MalariaGEN resource? It wasn't clear.

This is the genome dataset from reference 29 (https://doi.org/10.1002/ece3.11103), which indeed includes genomes from the MalariaGen resource, but supplemented with genomes from our own and our collaborators studies in Latin America, as well as other more recent publications. We have rephrased the sentence in lines 297-300 to clarify which dataset we are referring to.

9. On p7, the authors refer to 753 loci, which are not mentioned in the abstract. Is this because they do not anticipate them to be consistently polymorphic? Some more details on these loci, such as their MAF profile or the allelic diversity of each amplicon would be helpful.

Indeed, the different SNP subsets were not clear, and clarification was added in the methods in lines 235-249 and in the results to address this particular comment in lines 305-308,

The Pv Peru-barcode was first evaluated in silico in a WGS dataset, using the barcode SNPs and a larger set of biallelic SNPs in the WGS data that were also present in the newly generated Pv AmpliSeq Peru data (n= 730). We used a subset of the WGS data to all biallelic SNPs in the Pv AmpliSeq v2 Peru target region, to have similar resolution for population structure achievable with the assay. In the WGS data not all the SNPs from the Pv AmpliSeq Peru (n=2909 in total) were present in this dataset, partly as the MAF filter applied in that dataset could have removed SNPs that are rarely observed in other regions in the world.

10. The legend for Figure 3 (and perhaps the text on p7) needs more explanation on what the regional groups (AFR, ESEA etc) refer to. These look like MalariaGEN definitions but there is no reference to the definitions.

Indeed, these are the classifications from Adam et al., 2022. We have added the reference to the figure legend (line 960).

11. The country clustering in Figure 3 is not very tight. Is the resolution similar with genomic data - perhaps a reference figure would help. Only Panama separates well, but I believe they have experienced a strong bottleneck as described in https://pubmed.ncbi.nlm.nih.gov/33315861/. Having said the above, I appreciate that DAPC will only show parts of the full data.

The country clustering is indeed ‘not as tight’ as with the whole WGS data (with 1,983,976 SNPs), we have added the PCA from the WGS paper as supplementary figure S2 Fig for comparison. However, with a much smaller number of SNPs (i.e. lower resolution) we do not expect such a strong country clustering in the PCA, which is also why we choose the smaller subset of SNPs instead of the full WGS data in Figure 3 B and D.

12. On p8, it's helpful to have read depth information but would be even more informative if we had more details on how many samples were multiplexed in each run as that can affect the yield and is an important cost consideration.

We usually multiplex 144 libraries in one run, this information was added to line 214. It is feasible to multiplex more samples in one run in terms of depth and quality, but the majority of the costs is in the library preparation and not the sequencing run, therefore it is not much more cost-efficient to increase the number of samples in one MiSeq run.

13. The Results did not describe any negative controls (human DNA or other Plasmodium Spp) - were these tested and, if so, how did they perform?

We did include these controls (see new supp fig S1), and they performed as expected. We have now included information on the negative controls used and the results obtained in lines 338-351.

14. On p8, the authors describe a lack of temporal trends with He (in the 41 SNPs) despite a trend with pi on the full variants - is this potentially because of how the 41 SNPs were selected? What years of collection, for example, do the genomic sets reflect? Or is the lack of a trend with He likely simply due to lower information content with the 41 SNPs?

Although we observe a trend in *He* (mean *He* decreases from 0.349 to 0.305) the difference is not significant. Therefore the lack of a significant trend is more likely due to the lower information content. Therefore, to address this question, we also calculated He over all biallelic SNPs and compared it to *pi* and *He* with only the barcode. By increasing the number of SNPs, the trend in *He* values across the years is significantly different (mean *He* decreases from 0.0378 in 2007/8 to 0.0251 in 2019/20, p < 2e-16). However, the *He* values with all biallelic SNPs are much lower than with the Peru barcode, because many biallelic variants have rare alternate alleles. We prefer to keep the figure and analysis as it is, with *He* with the barcode SNPs and *pi* with all variants.

15. On p8, can the authors clarify whether Fst was run on the 41 SNPs or all variants?

Fst was calculated with all biallelic SNPs, and we clarified this in lines 263-264.

16. Was IBD calculated on the 41 SNPs or all variants? If the 41 SNPs, a comment in regards the 2019 study by Taylor mentioned above would be helpful.

We used all biallelic SNPs detected with the AMpliSeq assay as input for IsoRelate, and after some additional filtering (now specified in lines 275-283) we retained 1219 SNPs to estimate pairwise IBD.

17. On p11, were the misclassified samples also heterozygote at any of the global barcode SNPs?

At all the loci in the misclassified samples, the genotypes are either homozygote or missing.

18. On p12, the authors refer to the utility of CT screening by qPCR - what CT was found to be useful in this study?

Ideally Ct values should be lower than 34 using Pv mtCOX1 qPCR protocol (https://doi.org/10.1186/s12936-018-2201-0), this was added to lines 597. The effect on parasite density on AmpliSeq results was explored in more detail in the Pv Vietnam version manuscript (<https://doi.org/10.3389/fcimb.2022.953187>)

We believe that the revisions have significantly strengthened the manuscript. We are grateful for the opportunity to revise and resubmit our work and are committed to ensuring that it meets the expectations of the journal's readership.

Thank you once again for considering our manuscript for publication. We appreciate your time and attention to our work and look forward to hearing from you regarding the outcome of the next phase of the review process.

On behalf of all authors,

Sincerely,

Eline Kattenberg & Anna Rosanas-Urgell
