# Supplementary material for: Plasmodium vivax genomic surveillance in the Peruvian Amazon with Pv AmpliSeq assay
Source: PLoS Negl Trop Dis. 2024 Jul 11;18(7):e0011879. doi: 10.1371/journal.pntd.0011879 (PMC11265702; doi:10.1371/journal.pntd.0011879)
Supplement: Peer review history

# Peer review: Decision Letter 0

**DOI:** 10.1371/journal.pntd.0011879.r001

**Authors:** Susan Madison-Antenucci, Andrés Aranda-Diaz

12 Apr 2024

Dear Dr. Kattenberg,

Thank you very much for submitting your manuscript "Plasmodium vivax genomic surveillance in the Peruvian Amazon with Pv AmpliSeq assay" for consideration at PLOS Neglected Tropical Diseases. As with all papers reviewed by the journal, your manuscript was reviewed by members of the editorial board and by several independent reviewers.

The reviewers agree that your manuscript meets the criteria for publication and have provided feedback to improve the clarity throughout the sections of the paper. I would like to highlight that added clarity on the sources of data for each of the figures and text and the conclusions derived from those analysis will ensure that the novel aspects of the tool presented in the manuscript are contrasted to previous work and available data, addressing some of the major comments from the reviewers. 

Based on the reviews, we are likely to accept this manuscript for publication, providing that you modify the manuscript according to the review recommendations. 

Please prepare and submit your revised manuscript within 30 days. If you anticipate any delay, please let us know the expected resubmission date by replying to this email. 

When you are ready to resubmit, please upload the following:

[1] A letter containing a detailed list of your responses to all review comments, and a description of the changes you have made in the manuscript. 

Please note while forming your response, if your article is accepted, you may have the opportunity to make the peer review history publicly available. The record will include editor decision letters (with reviews) and your responses to reviewer comments. If eligible, we will contact you to opt in or out

[2] Two versions of the revised manuscript: one with either highlights or tracked changes denoting where the text has been changed; the other a clean version (uploaded as the manuscript file).

Important additional instructions are given below your reviewer comments. 

Thank you again for your submission to our journal. We hope that our editorial process has been constructive so far, and we welcome your feedback at any time. Please don't hesitate to contact us if you have any questions or comments.

Sincerely,

Andrés Aranda-Diaz, Ph.D.

Guest Editor

PLOS Neglected Tropical Diseases

Susan Madison-Antenucci

Section Editor

PLOS Neglected Tropical Diseases

***********************

Reviewer's Responses to Questions

**Key Review Criteria Required for Acceptance?**

As you describe the new analyses required for acceptance, please consider the following:

**Methods**

-Are the objectives of the study clearly articulated with a clear testable hypothesis stated?

-Is the study design appropriate to address the stated objectives?

-Is the population clearly described and appropriate for the hypothesis being tested?

-Is the sample size sufficient to ensure adequate power to address the hypothesis being tested?

-Were correct statistical analysis used to support conclusions?

-Are there concerns about ethical or regulatory requirements being met?

Reviewer #1: Number of samples you attend vs successfully genotyped not mentioned. This is very important to see your new Pv AmpliSeq panel robustness. Moreover, the following statement “For the analysis of genomic surveillance use cases in Peru, we included 230 samples with good quality data (<25% missing genotype calls for all variants, mean coverage >15), and retained only one library of replicates (with lowest missingness)” not clear. What is minimum sample and SNPs missingness rate in your data set. How many of samples and SNPs/loci/genotypes removed from total based on your filtering criteria. The association between sequence coverage and parasite density is critical but not mentioned!

Reviewer #2: -Are the objectives of the study clearly articulated with a clear testable hypothesis stated?

Yes

-Is the study design appropriate to address the stated objectives?

Yes

-Is the population clearly described and appropriate for the hypothesis being tested?

Yes

-Is the sample size sufficient to ensure adequate power to address the hypothesis being tested?

Yes

-Were correct statistical analysis used to support conclusions?

Yes

-Are there concerns about ethical or regulatory requirements being met?

No

Reviewer #3: Yes

--------------------

**Results**

-Does the analysis presented match the analysis plan?

-Are the results clearly and completely presented?

-Are the figures (Tables, Images) of sufficient quality for clarity?

Reviewer #1: Yes, well addressed. Few comments attached below.

Reviewer #2: -Does the analysis presented match the analysis plan?

Yes

-Are the results clearly and completely presented?

Yes

-Are the figures (Tables, Images) of sufficient quality for clarity?

They seem good but too low res to conclude now. Labeling throughout does seem limited and small in font.

Reviewer #3: Yes - mostly, just some minor clarifications needed as described in the report

--------------------

**Conclusions**

-Are the conclusions supported by the data presented?

-Are the limitations of analysis clearly described?

-Do the authors discuss how these data can be helpful to advance our understanding of the topic under study?

-Is public health relevance addressed?

Reviewer #1: Yes. Here are some comments. From FST vs IBD sharing and HE VS FWS. Worthy to mention which metrics are robust for Pv genomic surveillance and more correlated with transmission intensity. Which metrics useful for malaria control efforts?

Reviewer #2: -Are the conclusions supported by the data presented?

Yes

-Are the limitations of analysis clearly described?

Yes

-Do the authors discuss how these data can be helpful to advance our understanding of the topic under study?

Yes

-Is public health relevance addressed?

Yes

Reviewer #3: Yes

--------------------

**Editorial and Data Presentation Modifications?**

Use this section for editorial suggestions as well as relatively minor modifications of existing data that would enhance clarity. If the only modifications needed are minor and/or editorial, you may wish to recommend “Minor Revision” or “Accept”. 

Reviewer #1: “Minor Revision”

Reviewer #2: Minor revision

Reviewer #3: Minor Revision

--------------------

**Summary and General Comments**

Use this section to provide overall comments, discuss strengths/weaknesses of the study, novelty, significance, general execution and scholarship. You may also include additional comments for the author, including concerns about dual publication, research ethics, or publication ethics. If requesting major revision, please articulate the new experiments that are needed.

Reviewer #1: This manuscript by Johanna Helena Kattenberg and colleagues examined Plasmodium vivax genomic surveillance in the Peruvian Amazon using multiplex and robust Pv AmpliSeq panel. The study is timely study as Pv highly prevalent in hard-to-reach community of Peru and it is a major impediment to achieve malaria elimination goals. Overall, the manuscript is well written however it lacks novelty and key gaps to be addressed by the study. Detecting previously reported population differentiation is not good enough.

Reviewer #2: Methods comments:

What was the success rate? You mention 230 samples, but how many did you attempt to use initially?

I think you could add a few more words on the in-house variant calling pipeline. Is this a (targeted) mapping + GATK haplotype calling procedure as commonly applied to WGS reads, or are additional amplicon denoising strategies applied?

Whats the target size range (excl adapters) and how many samples do you sequence in single 600-cycle run? 

Why do you select Fws ≥ 0.95 as a proxy for a monoclonal infection?

Would it be useful to show sampling month in Fig 2 (e.g., as pie slices) and in S1 Table, since suggesting outbreak dynamics in some areas of text?

Results comments:

Fig. 1A: Could be expanded with larger labels and replace 1B,C,D - which currently provide little extra info. 

Since text talks of peri-urban Pv connectivity (as opposed to simply spatial proximity-based connectivity) it would be useful to see some representation of urban extent (around Iquitos etc) in the map. Currently also the grey lines are not clearly explained. Rivers etc? Also, it could be useful to use some of the same province-level coloring in components of Figs 5-6? (since it seems most relatedness patterns related to within versus between province membership).

Fig. 3: I suggest to label the genome/assay regions underlying each PCA directly in the subplots.

Figs 5A-B: Currently the node colors are not visible enough. Could make them bigger and better associate coloring with geography, e.g., add a small map and/or associate coloring with main map in Fig. 1.

How are p-values calculated for differences in Pi for Iquitos compared to its surrounding districts and for 2007-2008 vs. later years?

Also please label Fig. 6A-B thresholds directly in the plot. 

Please clarify meaning of left vs. right in Fig. 6C. Thickness of curves on the left indicates how many samples from the left-indicated district show IBD > 0.10 IBD to any sample in the district indicated at right? Etc.

Conclusions/Discussion comments:

I think a few sentences putting results in the context of transmission dynamics observed in other South American Pv regions would be useful. For example, the study suggests relatively clear regional clustering within Peru, signals of Brazilian diversity and outbreaking in Mariscal Ramon Castilla, and temporal turnover in Maynas. Do other regions show similar degrees of spatiotemporal population differentiation? If yes/no, how might environmental configuration and human activity patterns play a role?

Reviewer #3: The article describes some great, new, country-specific (Peru) amplicon-based sequencing tools for P. vivax that have the potential to support control and elimination efforts in Peru. The methods, the data showcasing the utilities of the tools and the conclusions are mostly robust. I recommend a few revisions that may help to improve the clarity of various approaches. One of the major confusions is regarding whether one can/should use the 41 biallelic SNPs and when one needs to use the full panel of variants called. The abstract only refers to 41 biallelic barcode SNPs, but many analyses use a larger panel of markers. More clarification on what one can expect from the tools (41 or hundreds of SNPs?) would be helpful for the reader to better understand what they might expect to achieve in their own studies if they use these tools. 

Minor revisions

1. The introduction has an entire paragraph describing the challenge of asymptomatic vivax in Peru (p3), which gives the implication that the tools can help to address this. However, the Discussion section doesn't revisit how the study tools address this particular challenge. Some comments on this would be helpful.

2. The Introduction also alludes to the asymptomatic infections as a P. vivax-specific challenge - is that strictly true?

3. On p4, AmpliSeq tools are described as having previously identified temporal challenges before and after NMCP intervention (along with other utilities) - are the appropriate references for this statement 22 and 23?

4. On p5, it would be helpful to better understand why the authors aimed for 49 SNPs? Is this purely because of the DAPC results or did they have a prior maximum number based on multiplex requirements with the other SNPs? Did they consider IBD requirements as per the specifications of Taylor and colleagues for 200 biallelic SNPs (https://pubmed.ncbi.nlm.nih.gov/31209105/)? Some further details would be helpful here. 

5. On p6, the authors describe <25% missingness as the cut-off - across all variants if I recall correctly. What was the maximum observed missingness at the 41-SNP barcode in the 230 defined good-quality samples?

6. On p6, the authors refer to 230 high-quality samples - is this 100% (i.e. 230/230) of samples on which sequencing was attempted?

7. On p7 the authors describe using Fws to characterize polyclonality - I believe on the 41-SNP barcode but they might clarify this. Given that the Fws was designed for genome-wide data, how well would this measure extrapolate to smaller barcodes? 

8. On p7, the authors refer to a genomic dataset comprising 1474 genomes - is this the MalariaGEN resource? It wasn't clear.

9. On p7, the authors refer to 753 loci, which are not mentioned in the abstract. Is this because they do not anticipate them to be consistently polymorphic? Some more details on these loci, such as their MAF profile or the allelic diversity of each amplicon would be helpful. 

10. The legend for Figure 3 (and perhaps the text on p7) needs more explanation on what the regional groups (AFR, ESEA etc) refer to. These look like MalariaGEN definitions but there is no reference to the definitions. 

11. The country clustering in Figure 3 is not very tight. Is the resolution similar with genomic data - perhaps a reference figure would help. Only Panama separates well, but I believe they have experienced a strong bottleneck as described in https://pubmed.ncbi.nlm.nih.gov/33315861/. Having said the above, I appreciate that DAPC will only show parts of the full data... 

12. On p8, it's helpful to have read depth information but would be even more informative if we had more details on how many samples were multiplexed in each run as that can affect the yield and is an important cost consideration.

13. The Results did not describe any negative controls (human DNA or other Plasmodium Spp) - were these tested and, if so, how did they perform?

14. On p8, the authors describe a lack of temporal trends with He (in the 41 SNPs) despite a trend with pi on the full variants - is this potentially because of how the 41 SNPs were selected? What years of collection, for example, do the genomic sets reflect? Or is the lack of a trend with He likely simply due to lower information content with the 41 SNPs?

15. On p8, can the authors clarify whether Fst was run on the 41 SNPs or all variants?

16. Was IBD calculated on the 41 SNPs or all variants? If the 41 SNPs, a comment in regards the 2019 study by Taylor mentioned above would be helpful.

17. On p11, were the misclassified samples also heterozygote at any of the global barcode SNPs?

18. On p12, the authors refer to the utility of CT screening by qPCR - what CT was found to be useful in this study?

--------------------

PLOS authors have the option to publish the peer review history of their article (what does this mean?). If published, this will include your full peer review and any attached files.

If you choose “no”, your identity will remain anonymous but your review may still be made public.

**Do you want your identity to be public for this peer review?** For information about this choice, including consent withdrawal, please see our Privacy Policy.

Reviewer #1: No

Reviewer #2: No

Reviewer #3: Yes: Sarah Auburn

Figure Files:

While revising your submission, please upload your figure files to the Preflight Analysis and Conversion Engine (PACE) digital diagnostic tool, https://pacev2.apexcovantage.com. PACE helps ensure that figures meet PLOS requirements. To use PACE, you must first register as a user. Then, login and navigate to the UPLOAD tab, where you will find detailed instructions on how to use the tool. If you encounter any issues or have any questions when using PACE, please email us at figures@plos.org.

Data Requirements:

Please note that, as a condition of publication, PLOS' data policy requires that you make available all data used to draw the conclusions outlined in your manuscript. Data must be deposited in an appropriate repository, included

Attachment Submitted filename: Reviewer_comments3.doc

# Author comment: Author response to Decision Letter 0

**DOI:** 10.1371/journal.pntd.0011879.r002

31 May 2024

Attachment Submitted filename: Reviewer_comments_and_responses_V2.docx

# Editor report: Decision Letter 1

**DOI:** 10.1371/journal.pntd.0011879.r003

**Authors:** Susan Madison-Antenucci, Andrés Aranda-Diaz

12 Jun 2024

Dear Dr. Kattenberg,

We are pleased to inform you that your manuscript 'Plasmodium vivax genomic surveillance in the Peruvian Amazon with Pv AmpliSeq assay' has been provisionally accepted for publication in PLOS Neglected Tropical Diseases.

Before your manuscript can be formally accepted you will need to complete some formatting changes, which you will receive in a follow up email. A member of our team will be in touch with a set of requests.

Please note that your manuscript will not be scheduled for publication until you have made the required changes, so a swift response is appreciated.

IMPORTANT: The editorial review process is now complete. PLOS will only permit corrections to spelling, formatting or significant scientific errors from this point onwards. Requests for major changes, or any which affect the scientific understanding of your work, will cause delays to the publication date of your manuscript.

Should you, your institution's press office or the journal office choose to press release your paper, you will automatically be opted out of early publication. We ask that you notify us now if you or your institution is planning to press release the article. All press must be co-ordinated with PLOS.

Thank you again for supporting Open Access publishing; we are looking forward to publishing your work in PLOS Neglected Tropical Diseases.

Best regards,

Andrés Aranda-Diaz, Ph.D.

Guest Editor

PLOS Neglected Tropical Diseases

Susan Madison-Antenucci

Section Editor

PLOS Neglected Tropical Diseases

***********************************************************

We have reviewed the revisions to your manuscript. The changes satisfactorily address the reviewers' concerns. Therefore, we are pleased to inform you that your manuscript has been accepted for publication.

# Editor report: Acceptance letter

**DOI:** 10.1371/journal.pntd.0011879.r004

**Authors:** Susan Madison-Antenucci, Andrés Aranda-Diaz

26 Jun 2024

Dear Dr. Kattenberg,

We are delighted to inform you that your manuscript, "*Plasmodium vivax* genomic surveillance in the Peruvian Amazon with Pv AmpliSeq assay," has been formally accepted for publication in PLOS Neglected Tropical Diseases.

We have now passed your article onto the PLOS Production Department who will complete the rest of the publication process. All authors will receive a confirmation email upon publication.

The corresponding author will soon be receiving a typeset proof for review, to ensure errors have not been introduced during production. Please review the PDF proof of your manuscript carefully, as this is the last chance to correct any scientific or type-setting errors. Please note that major changes, or those which affect the scientific understanding of the work, will likely cause delays to the publication date of your manuscript. Note: Proofs for Front Matter articles (Editorial, Viewpoint, Symposium, Review, etc...) are generated on a different schedule and may not be made available as quickly.

Soon after your final files are uploaded, the early version of your manuscript will be published online unless you opted out of this process. The date of the early version will be your article's publication date. The final article will be published to the same URL, and all versions of the paper will be accessible to readers.

Thank you again for supporting open-access publishing; we are looking forward to publishing your work in PLOS Neglected Tropical Diseases. 

Best regards,

Shaden Kamhawi

co-Editor-in-Chief

PLOS Neglected Tropical Diseases

Paul Brindley

co-Editor-in-Chief

PLOS Neglected Tropical Diseases
